# FBI-1 enhanced the resistance of triple-negative breast cancer cells to chemotherapeutic agents via the miR-30c/PXR axis

**DOI:** 10.1038/s41419-020-03053-0

**Published:** 2020-10-13

**Authors:** Hua Yang, Lili Ren, Yanan Wang, Xuebing Bi, Xiaoli Li, Ming Wen, Qian Zhang, Yang Yang, Youchao Jia, Yumiao Li, Aimin Zang, Yaning wei, Guanghai Dai

**Affiliations:** 1 Department of the Medical Oncology, the PLA General Hospital, Beijing, 100853 China; 2 Department of the Medical Oncology/the Hebei Key Laboratory of the Cancer Radiotherapy and Chemotherapy, Baoding City, 071000 Hebei province P.R. China; 3 Department of the Gastrointestinal Surgery, the Affiliated Hospital of Hebei University, Baoding City, 071000 Hebei province P.R. China

**Keywords:** Breast cancer, Breast cancer

## Abstract

The factor that binds to the inducer of short transcripts‐1 (FBI-1) is a transcription suppressor and an important proto‐oncogene that plays multiple roles in carcinogenesis and therapeutic resistance. In the present work, our results indicated that FBI-1 enhanced the resistance of triple-negative breast cancer (TNBC) cells to chemotherapeutic agents by repressing the expression of micoRNA-30c targeting the pregnane X receptor (PXR). The expression of FBI-1 was positively related to PXR and its downstream drug resistance-related genes in TNBC tissues. FBI-1 enhanced the expression of PXR and enhanced the activation of the PXR pathway. The miR-30c decreased the expression of PXR by targeting the 3′-UTR of PXR, and FBI-1 increased the expression of PXR by repressing miR-30c’s expression. Through the miR-30c/PXR axis, FBI-1 accelerated the clearance or elimination of antitumor agents in TNBC cells (the TNBC cell lines or the patients derived cells [PDCs]) and induced the resistance of cells to antitumor agents. Therefore, the results indicated that the miR-30c/PXR axis participates in the FBI-1-mediated drug-resistance of TNBC cells.

## Introduction

FBI‐1 (factor that binds to inducer of short transcripts‐1), also named leukemia/lymphoma‐related factor (LRF), osteoclast‐derived zinc finger (OCZF), Pokemon, or Zinc finger and BTB domain-containing protein 7A (ZBTB7A), has been considered an important proto‐oncogene and plays important roles in human malignancies^1–3^. FBI-1 can not only promote the proliferation or metastasis of human cancer cells (HCC) by repressing tumor suppressors but can also induce the resistance of HCC cells to antitumor agents. Multiple pieces of evidence have revealed that FBI-1 is overexpressed in several human cancers, including lung cancer, hepatocellular carcinoma, breast cancer, and colorectal cancer^4–7^. Therefore, FBI-1 is a promising and valuable target for human malignancy treatment.

Breast cancer is still the foremost fatal threat of human malignancy to female health^8,9^. Breast cancer can be divided into endocrine-dependent breast cancer, HER2-positive breast cancer, and triple-negative breast cancer (TNBC), according to its histological characteristics^10–12^. At present, endocrine-dependent breast cancer and HER2-positive breast cancer have drugs for symptomatic relief (e.g., Tamoxifen and Trastuzumab, respectively), and patients have a relatively good prognosis, while TNBC patients have strong heterogeneity^11–15^. TNBC treatments have limited efficacy and poor prognosis, especially compared to the other two kinds of breast cancer^11–13^. Therefore, it is of great significance to study and discover new intervention targets for anti-tumor treatment of TNBC and to research and develop more effective anti-tumor treatment strategies for this kind of cancer. Increasing evidence has indicated that TNBC has heterogeneity, and olaparib, an inhibitor of PARP (poly ADP-ribose polymerase), could be used in treating TNBC patients with a BRCA mutation^16,17^. However, work should be done to achieve more effective olaparib treatment for TNBC.

pregnane X receptor (PXR) can not only act as a central regulator for the metabolism and detoxification for exogenous drugs or toxicants in liver organs, but can also promote metabolism and clearance of chemotherapeutic drugs by mediating the expression of its downstream drug resistance-related genes in HCCs^18–20^. In the past, researchers have focused on the role of PXR in the liver and HCC. Recent studies have shown that PXR also plays an important role in other malignancies, including gastric, colorectal, and especially breast cancer^18–20^. Our previous work (the not published data) wish to extend our knowledge of FBI-1 in TNBC chemoresistance and found that knockdown of FBI-1 could repress the expression of *abcb1 (mdr-1)*, an important regulator of multi-drug resistance encoding P-glycoprotein (P-GP), a typical downstream gene of PXR. Based on these results, we hypothesize that FBI-1 would influence the activation of PXR pathway. Results of this work have indicated that FBI-1 enhanced the expression of PXR and the activation of the PXR pathway to accelerate the clearance or elimination of antitumor agent olaparib in TNBC cells (the TNBC cell lines or PDCs); it also induced the resistance of cells to olaparib. FBI-1 increased the expression of PXR by repressing the expression of miR-30c, which targeted the 3′-untranslated region (UTR) of PXR.

## Materials and methods

### Patients and agents

The use of clinical specimens was approved by the ethics committee of the Chinese PLA General Hospital, Beijing, China. A total number of 30 paired TNBC (subtype with BRCA mutation) clinical specimens (TNBC tissues or paired non-tumor tissues) were obtained via daily surgical resection with written information of patients collected from 10 May 2017 to 6 March 2019. All the protocols or experiments were performed according to the Helsinki Declaration. The sample size (30 paired nontumor/tumor specimens) used in the present work has adequate power to detect a pre-specified effect size (the 1-β: 0.8; α/2: 0.025; *P* < 0.05). The original hypothesis was that the expression level of the targeting gene (for example, FBI-1) was not significantly different in the non-tumor tissues compared with the tumor tissues; whereas the alternative hypothesis was that the expression level of the targeting gene was significantly different in the non-tumor tissue compared with the tumor tissue. The lentivirus particles containing the full-length sequence of the FBI-1 or the miR-30c (pre-miR-30c) were purchased from Vigene Corporation, Jinan City, Shandong Province, China. The lentivirus particles containing the full-length sequences of PXR or PXR with mutated miR-30c binding sites were purchased and prepared by Vigene Corporation, Jinan City, Shandong Province, China. The antitumor agent, olaparib (S1060), was purchased from Selleck Corporation, Houston, Texas, USA. For the cell-based experiments, antitumor agents were dissolved in DMSO and diluted by DMEM with 5% FBS. For the animal experiments, the agents were dissolved using PEG400 or Tween80 and dissolved by physiological saline. The plasmids, the luciferase reporters of PXR’s downstream genes (XREM-Luc, PXRE-Luc, DR3-Luc, or ER6-Luc), were a gift from Dr. Fan Feng in the Fifth Medical Center, Chinese PLA General Hospital^19–21^. The promoter region sequences of miR-30c’s promoter were obtained by chemical synthesis and cloned into pGL4.26 vectors (Origin Corporation, USA).

### The cell lines of TNBC

The cell lines of TNBC used in the present work including current TNBC cell lines and the patients-derived cells (PDCs). For the current TNBC cell lines, HCC-1937 and MDA-MB-436, which carry mutated BRCA1 were gift from prof. Qinong Ye in Department of Medical Molecular Biology, Beijing Institute of Biotechnology, Collaborative Innovation Center for Cancer Medicine, 100850, Beijing, China. For the PDCs, ten lines of PDCs were conserved in our lab and prepared following the methods descripted by Zhang et al.^21^. In brief, the pathological methods were used to determine the type of breast cancer tissue (TNBC) and the mutation-features of BRCA1. On this basis, the TNBC tumor tissues were collected and DMEM supplemented with 20% FBS was using on a sterilized 200-mesh steel sieve to obtain a suspension of TNBC cells by grinding softly. Then, the cell suspension was washed for twice with DMEM supplemented with 20% FBS (centrifuge the cell suspension at 600 rpm for 3 min, re-suspend it in DMEM and then centrifuge at 600 rpm for 3 min) to obtain TNBC PDCs. TNBC cells (the cell lines or PDCs) were conserved in liquid nitrogen for long-term storage, and were cultured in DMEM + 10% FBS at 37 °C and 5% CO_2_ condition.

### Correlation between FBI-1 and factors of PXR pathway in TNBC clinical tissues

The expression level of FBI-1, PXR (NR1I2), ABCG2/BCRP, or ABCB1/MDR-1 in TNBC clinical tissues was examined by quantitative polymerase chain reaction (qPCR) methods^22^. The correlation between FBI-1 and PXR, ABCG2, or MDR-1 was examined by SPSS software (version number 9.0, IBM, USA). The results were shown as scatter-plot images, the equation of correlation-analysis, or the *P*-Value.

### Cell-survival examination

The TNBC cells were treated with the indicated concentration of agents (10, 3, 1, 0.3, 0.1, 0.03, or 0.01 μmol/L for olaparib) for 48 h. Then, cells were analyzed by the Sulforhodamine B (SRB) assay, following the methods provided by and Li et al.^23^ and Guan et al.^24^. The relative survival cells were determined by the O.D. values (optical density values) at 510 nm. The inhibition rates were calculated as (*A*_510nm-control_ of 48 h − *A*_510nm-compound_ of 48 h)/(*A*_510nm-control_ of 48 h − *A*_510nm-control_ of 0 h) * 100^23,24^. The IC_50_ values of agents were calculated from the inhibition rates^23,24^. The colony formation experiments were performed following the method desicripted by Fang et al.^5^. TNBC cells were transfected with the plasmids and treated with solvent control or the olaparib. The cells were analyzed by the colony-formation.

### Luciferase experiments

The transcription factor activation of PXR was examined by the activation of luciferase reporters. PXR only contains AF-2 in the LBD domain, which makes it only have ligand-dependent transcription factor activity, and its activity depends on the role of the ligand. Rifampicin is the most typical and representative ligand/agonist of PXR. A series of concentration gradients of Rifampicin can induce the activity of the Luciferase reporter gene in the promoter region of its downstream genes or induce the transcription of PXR downstream genes in a dose-dependent manner. The TNBC cells, which were transfected with luciferase reporters or plasmids, were treated with the indicated concentration (10, 3, 1, 0.3, 0.1, 0.03, or 0.01 μmol/L) of rifampicin (Cat. No.: S1764, Selleck Corporation, USA), a typical agonist of PXR. After treatment of rifampicin for 24 h, TNBC cells were harvested for luciferase activation, which was performed using kits purchased from the Promega Corporation (Madison, Wisconsin, USA) following the manufacturer’s instruction or the methods described by Yang et al. and Lu et al.^25,26^. The EC_50_ values of rifampicin in each group were calculated using luciferase activation.

### Quantitative polymerase chain reaction (qPCR)

The total RNA samples were extracted from TNBC cells or subcutaneous tumor tissues, and the mRNA was subjected to reverse transcription. The reverse transcription and qPCR experiments were performed in accordance with methods described by Wang et al. and Ma et al.^27,28^. The primers used in the qPCR experiments were listed as (1) Pokemon (ZBTB7A), Forward Sequence 5′-GCAACATCTGCAAGGTCCGCTT-3′, Reverse sequence 5′-TCTTCAGGTC GTAGTTGTGGGC-3′; (2) PXR (NR1I2), Forward sequence 5′-GGACCAGCTGCAGGAGCAAT-3′, Reverse sequence 5′-CATGAGGGGCGTAGCAAAGG-3′; (3) MDR-1 (ABCB1) Forward sequence 5′-GCTGTCAAGGAAGCCAATGCCT-3′, Reverse sequence 5′-TGCAATG GCGATCCTCTGCTTC-3′; (4) ABCG2 (BCRP): Forward sequence 5′-GTTCTCAGCAGCTCTTCGGCTT-3′, Reverse sequence 5′-TCCTCCAGACACACCACGGATA-3′; (5) β-actin Forward sequence 5′-CACCATTGGCAATGAGCGGTTC-3′, Reverse sequence 5′-AGGTCTTTGCGGAT GTCCACGT-3′.

### Western blot examination

Cells were transfected with plasmids, and the total protein samples were extracted from cells or subcutaneous tumor tissues. The protein samples were used for the western blot experiments following the methods described by Wang et al.^29^ and Li et al.^30^. The protein levels of FBI-1 (ab175918), PXR (ab85451), ABCG2/BCRP (ab130244), and P-GP (ab140549) were examined by their antibodies (Abcam Corporation, Cambridge, CB2 0AX, UK), and the β-Actin was used as the loading control.

### Co-immunoprecipitation (co-IP) experiments and chromatin immunoprecipitation (ChIP) experiments

TNBC PDC No. 6 (TNBC PDC with high level of PXR) or TNBC PDC No. 3 (TNBC with low level of PXR) was transfected with a FLAG vector or the FLAG-FBI1 vector. Then, cells were harvested for IP (co-immunoprecipitation) experiments. Forty-eight hours after transfection, the cells were lysised, and the FLAG-FBI-1 was separated from the system with beads connected with the mono-clone FLAG antibody; the HA-PXR was examined using the mono-clone HA antibodies (Sigma-Aldrich, Merck KGaA, Darmstadt, Germany) via western blot experiments^31^. The ChIP was performed following the methods descripted in the previous publications^19–21^. The Protein-DNA complex (FBI-1, SP1 or PXR with DNA fragment) was separated by using their antibodies (anti-SP1, Cat. No.: ab231778, Abcam Corporation, UK). The IgG was used as the negative control for the antibodies. The recruitment of PXR to *mdr-1*’s enhancer region (Forward primer: 5′-GCAGTGTTT CTTGTATATGG-3′; Reverse primer: 5′-CTCAAATGAACTCTCTCC-3′) or the recruitment of SP1 to miR-30c’s promoter (fragment No. 1, Forward primer: 5′-TAAAGTTGAGCAAGTG CC-3′; Reverse primer: 5′-CCTCAACTACCTCCTACC-3′; fragment No. 2, Forward primer: 5′-GTCCTAACAACACAAACCT-3′; Reverse primer: 5′-CCCTTTAAAAACCCCTTCC-3′; fragment No. 3, Forward primer: 5′-GGGATAACTGGAGACTAA-3’; Reverse primer: 5′-CCAAGAAACAGAAGCCAA-3′; fragment No. 4, Forward primer: 5′-GGGGTTG AAATTGTTGTG-3′; Reverse primer: 5′-GGTTGATATAGTCTGTGCTT-3′; fragment No. 5, Forward primer: 5′-CATCATTCATCACGCACTT-3′; Reverse primer: 5’-TATTGAC CCCATCCCCAC-3′; fragment No. 6, Forward primer: 5′-ACATAGTGTGGGGATGGG-3′; Reverse primer: 5′-GGGCTGGCTGAGTAAAAA-3′) was examined. The negative genome sequence was chosen as the negative control for the recruitment-examination (Forward primer: 5′-AACCTATTAACT CACCCTTGT-3′; Reverse primer: 5′-CCTCCATTCAAAAGAT CTTATTATTTAGCATCTCCT-3′)^32^.

### Pharmacokinetic experiments

TNBC cells transfected with plasmids were used for the pharmacokinetic experiments to examine the amount of olaparib sustained in the cells. For the cell-based experiments, cells were treated with 1 μmol/L concentration of olaparib for 12 h^33^. Then, cells were harvested at indicated time-points. For the animal experiments, TNBC cells were injected into the subcutaneous position to form subcutaneous tumor tissues. After 2–3 weeks’ growth, when the tumor volumes reached 1500–2000 mm^3^, the solution of olaparib was injected into the subcutaneous tumors. Then, the tumor tissues were harvested at indicated times^34,35^. The olaparib in cells or tumor tissues was extracted by acetonitrile (ACN), and the amount of olaparib sustained in cells or tumor tissues at each time point was determined by liquid chromatography–mass spectrometry/mass spectrometry (LC-MS/MS), following the methods established by Krens et al.^36^. The sustaining rates of olaparib were calculated as (the amount of olaparib at each time)/(the amount of olaparib at 0-time) × 100%. The sustaining curves or the half-time life (t_1/2_) of olaparib was obtained by the sustained rates of olaparib.

### The in vivo antitumor effect of olaparib

Female nude mice, 4–6 weeks old, were purchased from Beijing Si-Bei-Fu Corporation, China. The animal experiments were approved by the Institutional Animal Care and Use Committee of the Chinese PLA General Hospital, China. All animal experiments (*n* = 10 for each group; animals were randomly divided into the groups) were performed according to the UK Animals (Scientific Procedures) Act, 1986, and associated guidelines. TNBC cells transfected with plasmids were treated with olaparib via oral administration once every two days. After 4–5 weeks’ treatment, the tumor tissues of the mice were harvested. The tumor volumes or tumor weights were determined following the methods described by Jia et al.^37^. The inhibition rates of olaparib were calculated by the tumor volumes or tumor weights^38^.

### Statistical analysis

All statistical significance analyses were performed using SPSS 9.0 statistical software (IBM Corporation, Armonk, NY, USA). The IC50 values or half-life time (t_1/2_) values were calculated by Origin software (Origin 6.1; OriginLab Corporation, Northampton, MA, USA). Statistical significance was analyzed by Bonferroni correction with two-way ANOVA, and paired samples were tested by paired-sample *t*-test (SPSS 9.0 statistical software; SPSS Inc., Chicago, IL, USA). All the images were quantitatively analyzed by the Image J software (National Institutes of Health [NIH], Bethesda, Maryland, USA).

## Results

### FBI-1 enhanced the activation of the PXR signaling pathway

**Fig. 1:**
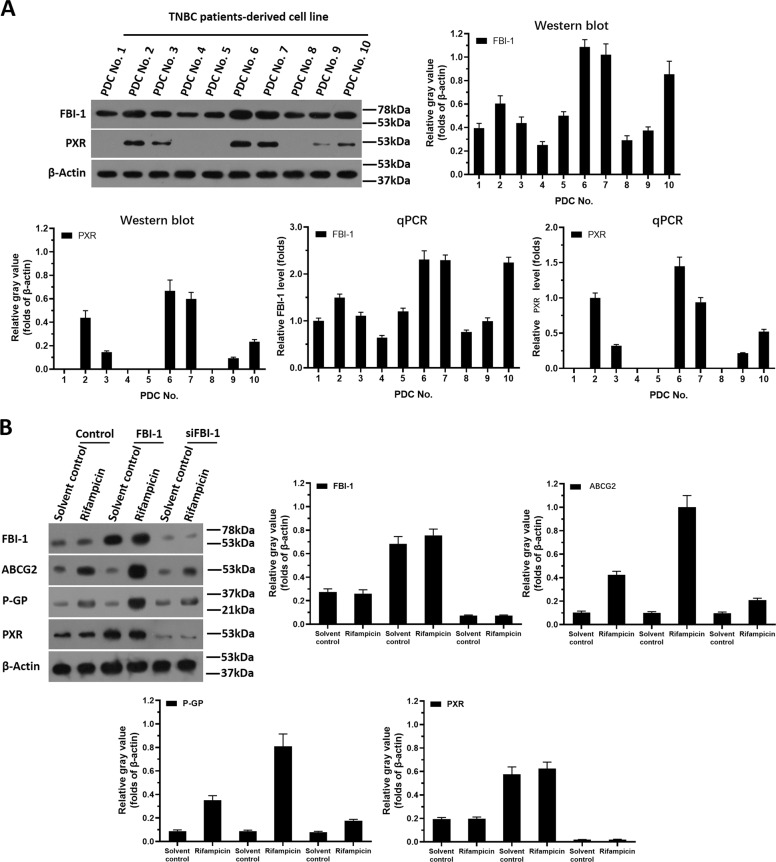
The expression of FBI-1 or PXR in patient-derived TNBC cells with mutated BRCA. **A** Ten lines of TNBC patient-derived cells (TNBC No. 1 to No. 10) were cultured and harvested for western blot experiments or qPCR. The endogenous protein levels of FBI-1 or PXR were examined by their antibodies and the results were shown as images of Western blot, quantitative analysis of images (mean ± SD) or the mRNA level of FBI-1 or PXR (mean ± SD). **B** FBI-1 enhanced the expression of protein levels in PXR’s downstream multi-drug resistance-related genes, BCRP and MDR-1, in patient-derived TNBC cells. TNBC PDC No. 3 was transfected with FBI-1 vector or the siRNA of FBI-1. The cells were cultured by using phenol red-free DMEM and activated carbon-treated serum (0.5%) and treated with 10μmol/L concentration of rifampicin. The protein samples were extracted and analyzed by western blot tests. The protein levels of FBI-1, BCRP, or P-GP were identified by their antibodies. The GAPDH was chosen as the loading control.

First, the expression of FBI-1 in TNBC clinical specimens was examined. As shown in Supplemental Fig. 1, the expression of FBI-1 and PXR pathway-related factors is much higher in TBNC specimens than their expression in the paired non-tumor tissues. Next, the protein expression of FBI-1 or PXR in patient-derived TNBC cells (PDCs) was examined. As shown in Fig. 1A, the expression of FBI-1 or PXR was detected in the ten lines of PDCs. Among these cell lines, PDC No. 3 with moderate expression of the FBI-1 or PXR was used to perform FBI-1 overexpression or FBI-1 knockdown. Moreover, PDCs No. 2, No. 6, or No. 7 with high level of endogenous FBI-1 or PXR were used to knock down the expression of FBI-1. The PDC No. 9 with the low level of endogenous FBI-1 or PXR was used to overexpress FBI-1.

**Fig. 2:**
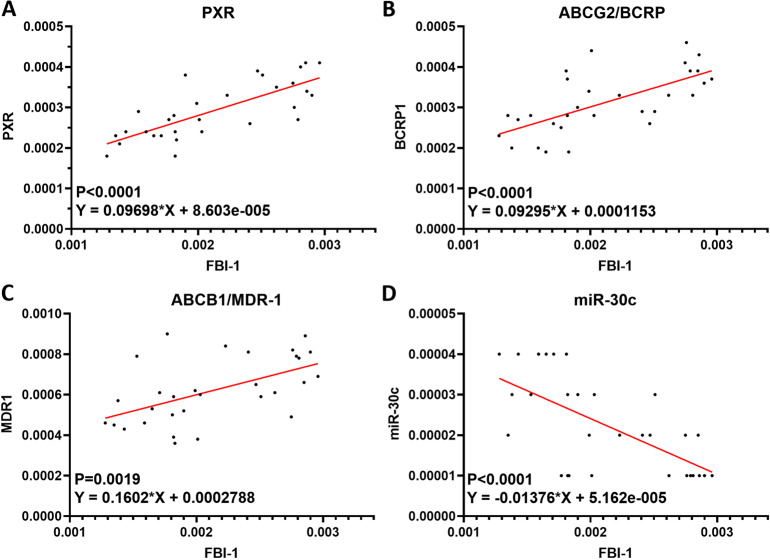
The association between FBI-1 and factors related to the PXR pathway. The mRNA level of PXR, BCRP, MDR-1, miR-30c, or FBI-1 in TNBC clinical specimens was examined using the qPCR experiments. The association between the expression of FBI-1 with PXR, BCRP, miR-30c, or MDR-1 was examined. The results were shown as scatter-plot images. The abscissa is the expression level of FBI-1; the ordinate is the corresponding expression level of PXR, BCRP, MDR-1, or miR-30c.

Next, the effect of FBI-1 on PXR’s activation was examined. The activation of PXR is dependent on the presence of its agonist/ligand and the transcription factor of PXR was examined by the EC_50_ values of luciferase reporters of PXR or the mRNA level of PXR’s downstream genes induced by rifampicin, a typical agonist/ligand of PXR. As shown in Table 1, overexpression of FBI-1 enhanced the activation of PXR (enhanced the activation of luciferase reporters and the mRNA level of *abcg2* or *mdr-1* induced by rifampicin), and the *EC*_*50*_ values of rifampicin were decreased. Knockdown of FBI-1 repressed the activation of PXR (repressed the activation of luciferase reporters and the mRNA level of *abcg2* or *mdr-1* induced by rifampicin), and the *EC*_*50*_ values of rifampicin were increased (Table 2). Next, As shown in Fig. 1B and Supplemental Fig. 2, overexpression of FBI-1 enhanced not only the protein level of PXR’s downstream drug resistance-related genes (ABCG2 [BCRP] or P-GP encoding by *mdr-1*), but also the expression of PXR; whereas knockdown of FBI-1 decreased not only the expression of PXR’s downstream drug resistance-related genes (ABCG2 [BCRP] or P-GP) but also the expression of PXR. Moreover, the relation between the mRNA expression of *zbtb7a/FBI-1* and the expression of *mdr-1* and *abcg2*, two typical PXR downstream gene was examined to support the effect of FBI-1 on PXR pathway. As shown in Fig. 2 and Supplemental Fig. 1B, *zbtb7a* was positively related with the expression of PXR, *abcg2*, or *abcb1/mdr-1* (Fig. 2 and Supplemental Fig. 1B).

**Table 1:** Overexpression of FBI-1 enhanced the transcription factor activation of PXR in TNBC cells.

Cell lines	Activation	Luciferase activation				mRNA level	
		XREN-Luc	PXRE-Luc	DR3-Luc	ER6-Luc	*Bcrp (abcg2)*	*mdr-1 (abcb1)*
		EC_50_ values of rifampicin (μmol/L)					
PDC No. 3	Control	6.55 ± 0.77	6.20 ± 0.42	7.05 ± 1.20	6.85 ± 1.55	5.37 ± 0.44	5.78 ± 0.39
	FBI-1	1.16 ± 0.50*	1.32 ± 0.54*	1.47 ± 0.25*	0.98 ± 0.18*	1.32 ± 0.09*	1.12 ± 0.07*
PDC No. 9	Control	N.A.	N.A.	N.A.	N.A.	8.40 ± 0.67	9.51 ± 2.73
	FBI-1	2.81 ± 0.47*	2.55 ± 0.87*	3.43 ± 1.77*	1.89 ± 0.72*	1.76 ± 0.54*	1.93 ± 0.65*

TNBC cells (PDC No. 3 and No. 9) which were transfected with plasmids were treated with indicated concentration of Rifampicin. The EC_50_ values of rifampicin (μmol/L) on the activation of luciferase reporters or the mRNA level of *mdr-1* or *bcrp* was shown as mean ± SD.

N.A. indicates the that the cells treated with a series of concentration of Rifampicin, and the agonistic activation of rifampicin in the control group’s cells was (for example, the maximum dose of the selected concentration of Rifampicin [10 μmol/L] has limited agonistic activity on these luciferase reporters or the mRNA level of PXR downstream genes). It is impossible to fit a dose-effect curve and to obtain the EC_50_ values.

*TNBC* triple-negative breast cancer, *PDC* patients-derived cells.

*P < 0.05 versus control group with FBI-1 group.

**Table 2:** Knockdown of FBI-1 decreased the transcription factor activation of PXR in TNBC cells.

Cell lines	Activation	EC_50_ values of rifampicin (μmol/L)					
		Luciferase activation				mRNA level	
		XREN-Luc	PXRE-Luc	DR3-Luc	ER6-Luc	*bcrp (abcg2)*	*mdr-1 (abcb1)*
PDC No. 3	Control	6.67 ± 1.01*	6.96 ± 0.86*	7.14 ± 1.16*	6.32 ± 0.25*	5.21 ± 0.72*	5.15 ± 0.30*
	siFBI-1	N.A.	N.A.	N.A.	N.A.	N.A.	N.A.
PDC No. 2	Control	5.41 ± 0.35*	5.60 ± 0.75*	5.39 ± 0.64*	6.25 ± 0.92*	4.42 ± 0.47*	3.97 ± 0.38*
	siFBI-1	N.A.	N.A.	N.A.	N.A.	N.A.	N.A.
PDC No. 6	Control	5.25 ± 0.60*	4.54 ± 0.44*	4.83 ± 0.95*	4.73 ± 0.79*	3.25 ± 0.66*	2.67 ± 0.50*
	siFBI-1	N.A.	N.A.	N.A.	N.A.	N.A.	N.A.
PDC No. 7	Control	4.87 ± 0.24*	4.62 ± 0.58*	5.44 ± 0.30*	3.15 ± 0.20*	2.89 ± 0.55*	3.41 ± 0.91*
	siFBI-1	N.A.	N.A.	N.A.	N.A.	N.A.	N.A.
PDC No. 9	Control	N.A.	N.A.	N.A.	N.A.	9.90 ± 0.38*	N.A.
	siFBI-1	N.A.	N.A.	N.A.	N.A.	N.A.	N.A.

TNBC cells (PDC No. 3, No. 2 No. 9) which were transfected with plasmids were treated with indicated concentration of Rifampicin. The EC_50_ values of rifampicin (μmol/L) on the activation of luciferase reporters or the mRNA level of *mdr-1* or *bcrp* was shown as mean ± SD.

N.A. indicates the that the cells treated with a series of concentration of Rifampicin, and the agonistic activation of rifampicin in the control group’s cells was (for example, the maximum dose of the selected concentration of Rifampicin [10 μmol/L] has limited agonistic activity on these luciferase reporters or the mRNA level of PXR downstream genes). It is impossible to fit a dose-effect curve and to obtain the EC_50_ values.

*TNBC* triple-negative breast cancer, *PDC* patients-derived cells.

*P < 0.05 versus control group with siFBI-1 group.

**Fig. 3:**
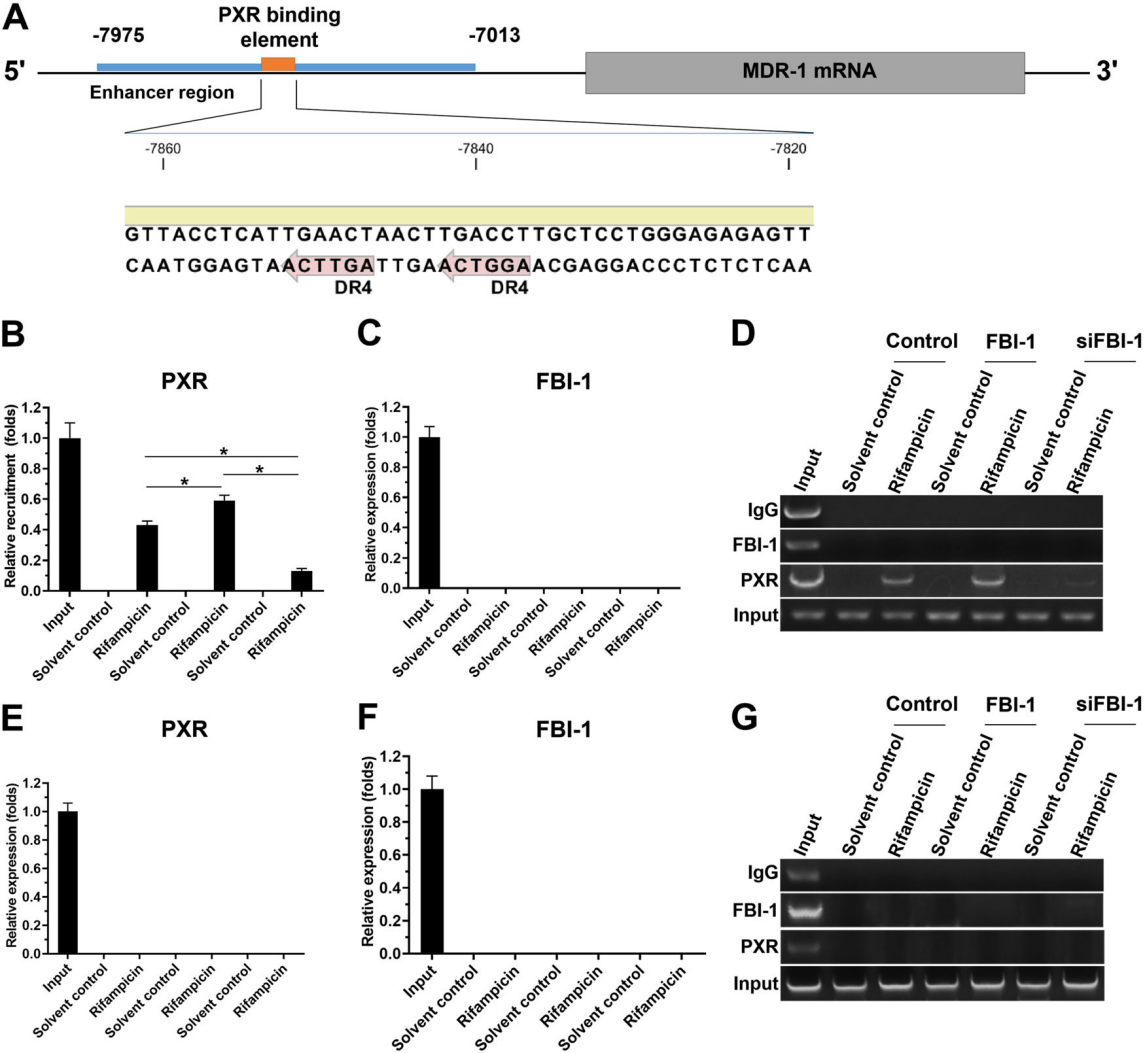
FBI-1 could not be recruited to the enhancer region of PXR’s downstream gene, *mdr-1*. **A** the binding site (DR-4) of PXR in the enhancer region of *mdr-1*. **B**–**G** TNBC PDC No. 2 was treated with rifampicin for 30 min to 1 h. Then, cells were harvested for the ChIP assays. The FBI-1 or PXR was separated from the system using their antibodies, and the DNA binding with the proteins identified through PCR that the enhancer region of *mdr-1* (**B**–**D**) or the negative genome region (**E**–**G**) was amplified. The results were shown as images from the DNA gel electrophoresis or the quantitative results (mean ± SD). *P < 0.05.

To further examine the specific effect of FBI-1 on the PXR pathway, the co-IP, ChIP, or western blot experiments were examined. As shown in Fig. 3, FBI-1 could enhance the recruitment of PXR to the enhancer region of its downstream gene (*abcb1*/*mdr-1*) which encoding the P-GP to enhance in the anti-tumor agent-resistance, however, it could not recruit itself to the promoter region of *mdr-1*. The protein-interaction results showed that FBI-1 did not interact with PXR (Supplemental Fig. 3). Therefore, FBI-1 enhanced the activation of the PXR pathway by enhancing the expression of PXR but not interacting with PXR.

### FBI-1 induced the resistance of TNBC cells to olaparib by accelerating elimination or clearance of olaparib

**Fig. 4:**
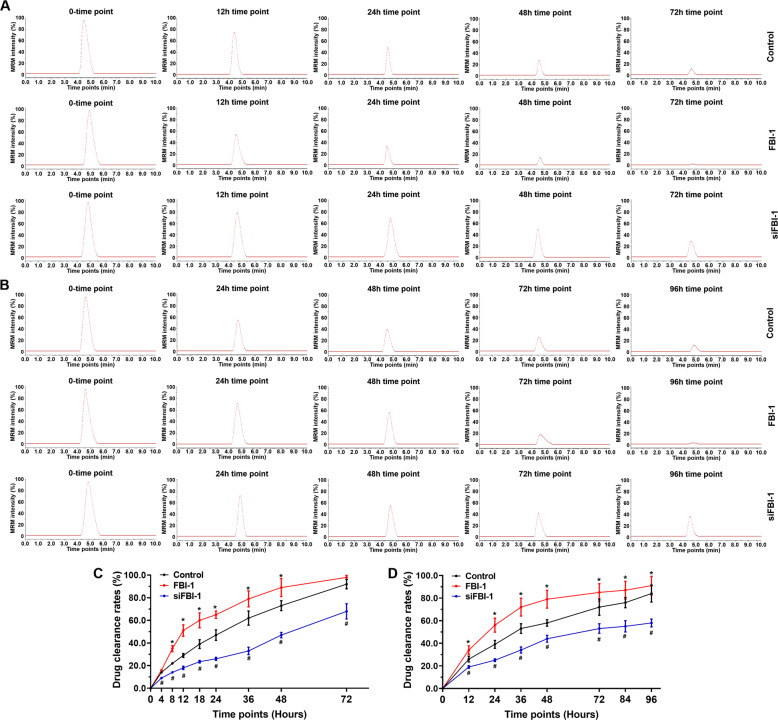
FBI-1 accelerated the elimination or clearance of olaparib in TNBC cells. **A**, **C** TNBC PDC No. 3 was cultured and treated with olaparib and harvested at the indicated time points. **B**, **D** TNBC PDC No. 1 was seeded into nude mice to form subcutaneous tumor models. The solution of olaparib was injected into the tumor tissues, which were harvested at indicated time points. The sustained amount of olaparib in the cultured cells (**A**) or the subcutaneous tumor tissues (**B**) was identified by the LC-MS/MS methods. The drug clearance rates were calculated as (the amount of olaparib at 0-time point) – (the amount of olaparib at the indicated time point) / (the amount of [] at 0-time point) * 100%. The drug-clearance curve was obtained based on the drug clearance rates. *P < 0.05 versus the control group with the FBI-1 overexpression group; #*P* < 0.05 versus the control group with the FBI-1 siRNA (knockdown) group.

The above data indicated that FBI-1 enhanced the activation of the PXR pathway. To further examine the effect of FBI-1, the elimination or the clearance of olaparib in TNBC cells was evaluated. As shown in Fig. 4 and Table 3, overexpression of FBI-1 accelerated the elimination or clearance of olaparib in TNBC cells, and the half-life time (t_1/2_) of olaparib decreased. The knockdown of FBI-1 decelerated the elimination or clearance of olaparib in TNBC cells, and the half-life time (t_1/2_) of olaparib increased. The results were shown as represented LC-MS/MS images or the sustaining curves of olaparib in No. 1 PDC and the half-life time (t_1/2_) of olaparib in TNBC cells (Table 3). Then, the specificity of FBI-1 was examined. Ketoconazole, a typical antagonist of PXR, was used. Moreover, as shown in Table 4, treatment of ketoconazole almost blocked the effect of FBI-1 on olaparib in TNBC cells. Therefore, FBI-1 accelerated the elimination or clearance of olaparib in TNBC cells by enhancing the activation of the PXR pathway.

**Table 3:** FBI-1 accelerated the clearance or the elimination of olaparib in TNBC cells.

Cell lines	Groups	Half life values of olaparib (t_1/2_, hours)	
		In cultured cells	In subcutaneous tumors
PDC No. 3	Control	25.03 ± 7.82	32.23 ± 9.21
	FBI-1	11.65 ± 3.88*	17.86 ± 8.65*
PDC No. 9	Control	33.75 ± 5.50	36.06 ± 8.33
	FBI-1	18.10 ± 4.44*	22.60 ± 4.36*
PDC No. 3	Control	25.03 ± 7.82	32.23 ± 9.21
	siFBI-1	59.25 ± 8.91*	62.72 ± 6.77*
PDC No. 2	Control	21.72 ± 6.89	30.14 ± 6.81
	siFBI-1	60.78 ± 4.85*	53.69 ± 7.34*
PDC No. 6	Control	19.43 ± 5.83	28.59 ± 9.84
	siFBI-1	51.35 ± 11.52*	43.64 ± 7.72*
PDC No. 7	Control	20.97 ± 5.01	31.41 ± 9.95
	siFBI-1	55.54 ± 9.97*	58.25 ± 9.06*

The TNBC cells (PDCs No. 3, 9, 2, 6, and 7) which were transfected with plasmid were treated with olaparib for 12 h. Then, cells were harvested and the amount of olaparib sustaining in cells were examined by the LC-MS/MS methods. The half-life values of olaparib were shown as the mean ± SD.

*TNBC* triple-negative breast cancer, *PDC* patients-derived cells, *LC-MS/MS* liquid chromatograph-mass spectrometer mass spectrometer.

*P < 0.05 versus control group with FBI-1 group.

*P < 0.05 versus control group with siFBI-1 group.

**Table 4:** Overexpression of FBI-1 enhanced the resistance of TNBC cells to olparib via PXR pathway.

Cell lines	Half-life values of olparib (t_1/2_, hours)		
	Control	FBI-1	FBI-1 + ketoconazole
PDC No. 3	27.38 ± 4.30	10.60 ± 4.54*	26.24 ± 3.85
PDC No. 9	36.33 ± 6.64	14.86 ± 2.88*	37.75 ± 5.55

The TNBC cells (PDCs No. 3 and 9) which were transfected with plasmid were treated with olaparib. The antitumor effect of olaparib on TNBC cells was shown as the IC_50_ values (mean ± SD).

*TNBC* triple-negative breast cancer, *PDC* patients-derived cells, *LC-MS/MS* liquid chromatograph-mass spectrometer mass spectrometer.

*P < 0.05 versus FBI-1 group with FBI-1 + ketoconazole group.

To examine the effect of FBI-1 on olaparib’s antitumor effect, the in vitro models were used. As shown in Supplemental Table 1 and Supplemental Table 2, overexpression of FBI-1 decreased the antitumor effect of olaparib on TNBC cells, and the IC_50_ values of olaparib were increased (Supplemental Table 1); knockdown of FBI-1 decreased the antitumor effect of olaparib on TNBC cells, and the IC_50_ values of olaparib were increased (Supplemental Table 2).

### FBI-1 repressed the expression of miR-30c by SP1

**Fig. 5:**
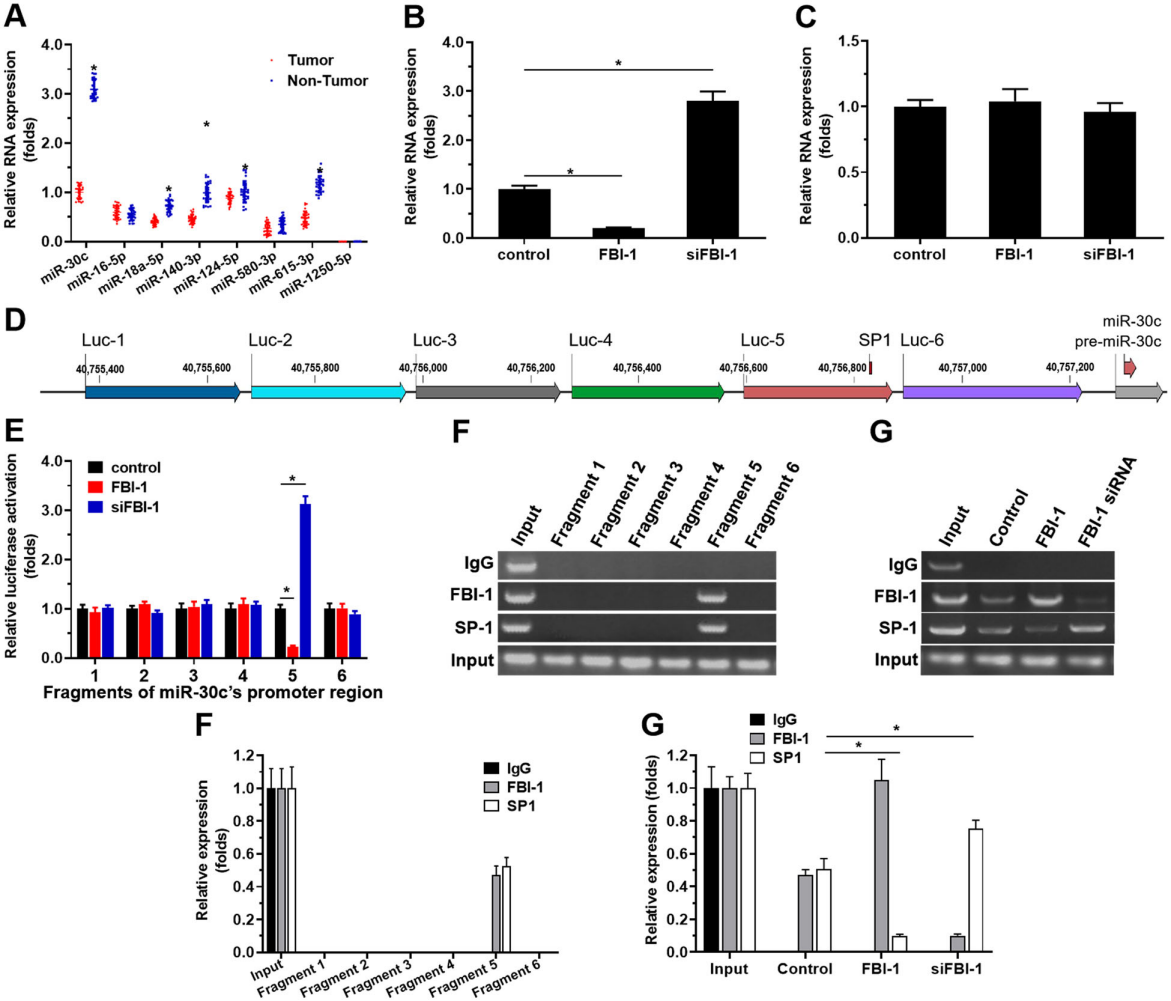
FBI-1 repressed the expression of miR-30c, which potentially targets PXR by repressing the recruitment of SP1 to miR-30c’s promoter region. **A** The miRs potentially targeting PXR was screened by an online tool, miRDB, and the expression level of miRs potentially targeting PXR was examined in the 30 paired TNBC specimens and the non-tumor tissues. **B**, **C** The effect of FBI-1 on the expression of miR-30c (**B**) or miR-140-3p was examined by qPCR. **D** The promoter region and the six luciferase reporters contain the six fragments of mR-30c’s promoter regions. **E** The effect of FBI-1 on the activation of the six luciferase reporters were examined by the luciferase-activation examination. **F**, **G** The recruitment of SP1 (**F**, **G**) or FBI-1 (**G**) to the promoter region of miR-30c was examined by the ChIP experiments. The quantitative results of (**F**) and (**G**) was shown as mean ± SD (**H**, **I**). *P < 0.05.

To examine the potential mechanisms of FBI-1 on PXR, the miRNA potentially targeting PXR was searched in PubMed and predicted by miRDB with high scores. The expression of these miRNAs in clinical specimens was examined. As shown in Fig. 5 and Supplemental Fig. 1A, the expression of miR-30c was highly expressed in the non-tumor tissues, compared with the paired TNBC tissues. Overexpression of FBI-1 decreased the expression of miR-30c, and knockdown of FBI-1 enhanced the expression of miR-30c. Moreover, miR-140-3p was another miRNA with a similar expressing feature to miR-30c, however, FBI-1 did not modulate the expression of miR-140-3p. The co-relation between PXR with miR-30c or miR-30c with FBI-1 further revealed the effect of FBI-1 on miR-30c on PXP pathway. As shown in Fig. 2 and Supplemental Fig. 1, the expression of FBI-1 was negatively related with miR-30c and the expression of miR-30c was also negative with PXR in clinical specimens. Thus, we chose miR-30c for the next steps of the study.

Next, to reveal why and how FBI-1 represses the expression of miR-30c, bioinformatics analysis was performed to reveal the features of the miR-30c’s promoter (http://tfbind.hgc.jp). As shown in Fig. 5 and Supplemental Fig. 4, miR-30c’s promoter contained a SP1 binding site. It has been confirmed that FBI-1 could repress the expression of targeting genes by interacting with SP1. The results indicated that overexpression of FBI-1 decreased the activity of the luciferase reporter containing the miR-30c promoter region with the SP1-binding site (luciferase reporter No. 5), but not the other regions (other luciferase reporters). Moreover, ChIP results indicated that FBI-1 could be recruited to the miR-30c’s promoter region containing the SP1-binding site and repress the recruitment of SP1 to this region. The ChIP results were confirmed by the luciferase analysis (Supplemental Fig. 4). FBI-1 repressed the activation of the luciferase reporter containing the promoter region of miR-30c with wild-type SP1 binding site but not the region did not contain SP1 binding site or containing the mutated SP1 binding site (Supplemental Fig. 4). These data suggest that FBI-1 could repress the expression of miR-30c by repressing the recruitment of SP1 to miR-30c’s promoter region.

### FBI-1 enhanced the expression of PXR via miR-30c

**Fig. 6:**
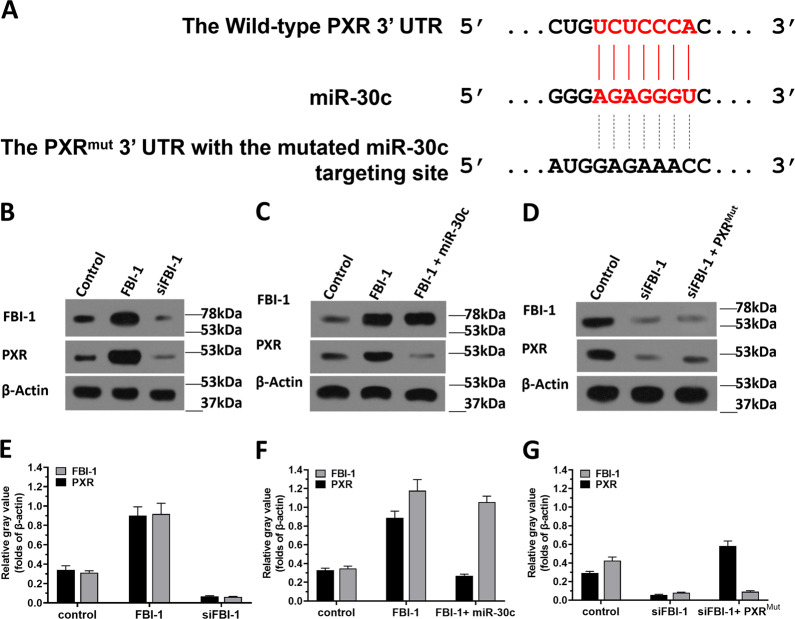
FBI-1 enhanced the expression of PXR via the SP1/miR-30c axis. **A** The targeting sites of miR-30c in PXR’s 3′UTR was shown as diagrammatic sketch. **B** TNBC PDC No. 1 was transfected with control, FBI-1, or siFBI-1. **C** TNBC PDC No. 1 was transfected with control, FBI-1, or FBI-1 + miR-30c. **D** TNBC PDC No. 1 was transfected with control, siFBI-1, or siFBI-1 + PXRMut. **B**–**D** The expression levels of FBI-1 or PXR were examined through the western blot experiments. The quantitative results of western blot images (**B**–**D**) were shown as mean ± SD (**E**–**G**).

Next, to further examine the effect of FBI-1 on miR-30c/PXR, the potential targeting sites of miR-30c in the 3′-UTR of PXR were shown in Fig. 6. Overexpression of FBI-1 enhanced the expression of PXR, and knockdown of FBI-1 via its siRNA (siFBI-1) decreased the expression of PXR. Overexpression of miR-30c inhibited the effect of FBI-1 overexpression to enhance the protein level of PXR; whereas siFBI-1 decreased the expression of PXR but not for PXR with the mutated miR-30c binding sites. The effect of FBI-1 on miR-30c/PXR was further confirmed by the colony-formation experiments and the expression level of related factors examined by qPCR (Supplemental Fig. 5). Therefore, FBI-1 enhanced the expression of PXR via miR-30c.

### The specificity of FBI-1’s function in regulating olaparib resistance in TNBC cells

**Fig. 7:**
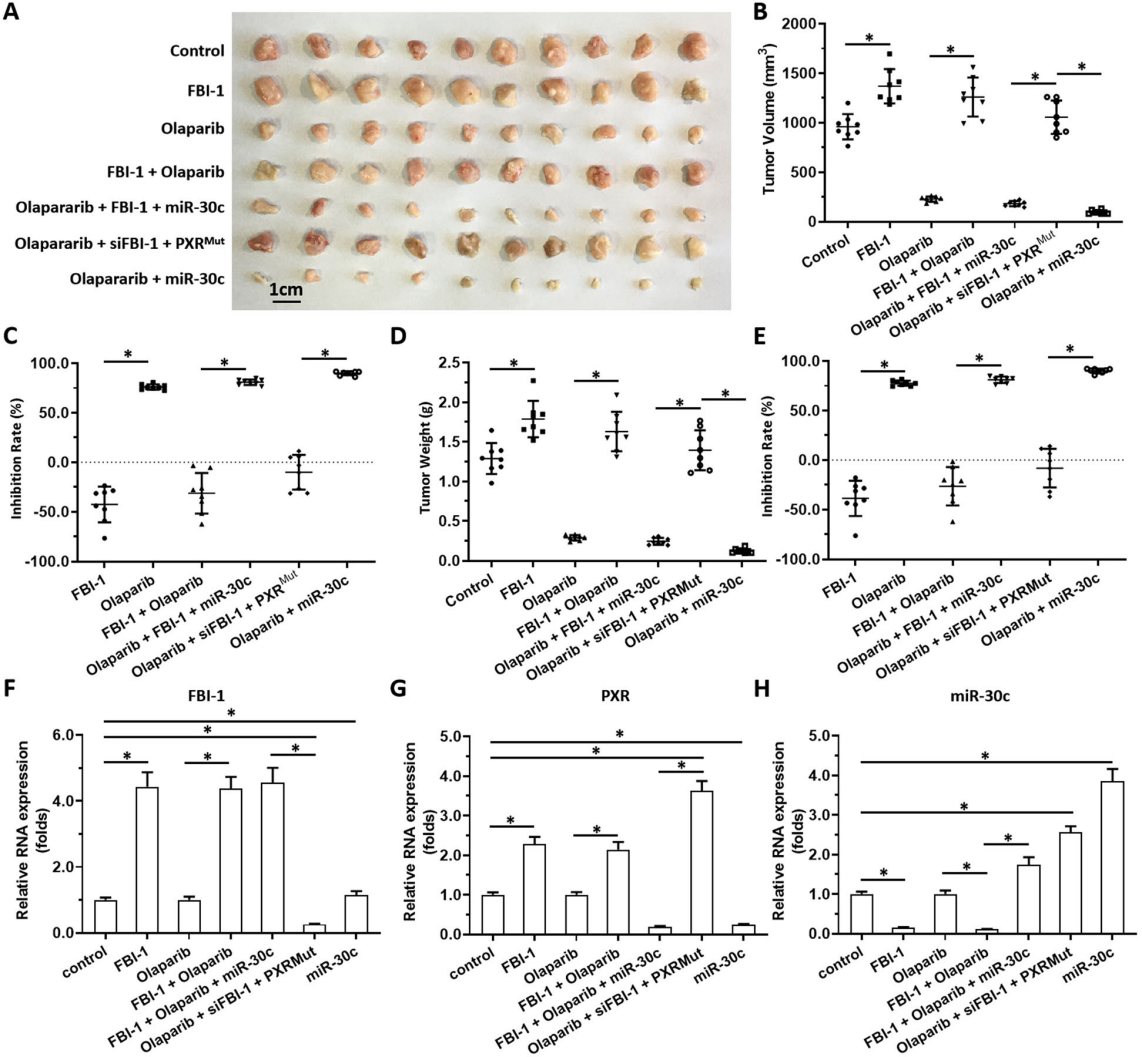
FBI-1 enhanced the resistance of TNBC cells to olaparib via the miR-30c/PXR axis. The TNBC cells (TNBC PDC No.1) was transfected with FBI-1, siFBI-1, miR-30c, or the PXRMut. Then, cells were injected into the subcutaneous position of nude mice to form tumor tissues. The images of tumor tissues were shown (**A**) and the tumor volumes or tumor weights, or the inhibition rates of olaparib according to tumor volumes or tumor weights, were shown as scatter-plot images (**B**–**E**). The expression of FBI-1 (**F**), PXR (**G**), or miR-30c (**H**) in tumor tissues was examined by qPCR and shown as a histogram (**F**–**H**). *P < 0.05.

The specificity of FBI-1 on olaparib’s antitumor effect was examined. As shown in Fig. 7, olaparib inhibited the subcutaneous growth of TNBC cells (TNBC PDC No. 3). Overexpression of FBI-1 enhanced the resistance of TNBC cells to olaparib. Overexpression of miR-30c not only enhanced the sensitivity of TNBC cells to olaparib with similar results to siFBI-1, but also overcame the resistance of TNBC cells to olaparib induced by FBI-1. Transfection of PXR with mutated miR-30c binding sites almost blocked the effects of siFBI-1 or miR-30c.

The above results were obtained from the PDCs of TNBC. To further examine the effect of FBI-1, the TNBC cell lines, HCC-1937 or MDA-MB-436, were used. As shown in Supplemental Figs. 6 and 7, FBI-1 enhanced the expression of PXR via miR-30c in HCC-1937 or MDA-MB-436 cells. Moreover, FBI-1 enhanced the resistance of HCC-1937 or MDA-MB-436 cells to olaparib and accelerated the metabolism or clearance of olaparib via miR-30c/PXR axis (Supplemental Tables 3 and 4). These results further confirmed the effect of FBI-1 on Therefore, FBI-1 enhanced the activation of olaparib via the miR-30c/PXR axis.

## Discussion

TNBC is a sub-type of breast cancer with ER-deficiency, HER2-decificiency, and PR-deficiency features^39,40^. Although the molecular targeting agents have been applied, the clinical outcomes and the prognosis of patients with TNBC are still poor^41,42^. Olaparib is a kind of molecular targeting agent (small molecular inhibitor) that targets the PARP and is used in treating TNBC with a BRCA mutation^43,44^. In the present work, the association of FBI-1 and the antitumor effect of olaparib in TNBC cells were considered. The single cells (PDCs) were separated from the clinical specimens of TNBC with BRCA-mutation. FBI-1 enhanced the resistance of TNBC PDCs to olaparib by accelerating its elimination or clearance in cells by the miR-30c/PXR axis. The results not only extended our knowledge of TNBC treatment but also provided clues that FBI-1 would be a promising target to achieve more effective TNBC treatment. Moreover, in patients suffering from TNBC with the positive expression of programmed death 1 (PD-1) in cancer cells or programmed death ligand 1 (PD-L1) occurring in tumor-infiltrating immune cells, the inhibition of PD1/PD-L1 may be a hopeful treatment strategy^9,10^. Combining atezolizumab, which is a selective antibody targeting PD-L1 to prevent the interaction between PD-1 and B7-1 and reverse T-cell suppression, with paclitaxel could prolong the progression-free survival of patients with metastatic TNBC’s PD-L1–positive subgroup^45,46^. The metabolic feature and the mechanisms of the therapeutic antibodies represented by Atezolizumab are much different from the small molecular kinase inhibitor. Therefore, work to achieve more effective treatment of therapeutic antibodies would be valuable in the future.

The downstream genes of PXR, such as P-GP, metabolize and eliminate antitumor drugs in tumor cells or tissues through various pathways, which can ultimately reduce the effective concentration of the drug in the tumor cells or tissues and finally induce the tumor cells to resist tumor drugs^47,48^. The regulation mechanism of PXR transcription factor activity has always been the focus of research. Drugs, including rifampicin, can act as PXR agonists to induce PXR transcription factor activity. Feng et al.^20^ indicated that during treatment, sorafenib could bind to PXR and function as an agonist of PXR to activate the cascade pathway by which HCCs develop sorafenib-resistance via accelerated elimination or clearance of sorafenib. In addition to agonists of PXR, co-regulators of PXR are also important factors affecting the activity of its transcription factors^21^. For example, ETS-1 or EPAS-1 can function as co-activators to up-regulate PXR activity^19–21^. In this study, FBI-1 was able to promote PXR expression by down-regulating miR and then up-regulating the PXR signaling pathway activity. The miRNA is a type of non-coding RNA transcribed by RNA Pol II. It can recognize and silence the expression of specific genes through specific sequences^49^^,50^. In the present work, miR-30c was identified as a kind of miR to enhance the sensitivity of TNBC cells to olaparib via PXR pathway. The two types of miR-30c (miR-30c-1 or miR-30c-2) could target the s3’UTR of PXR (Supplemental Fig. 8). The results of this study show that FBI-1-mediated silencing of miRNA expression may be a possible cause of PXR overexpression in TNBC cells. As a transcription inhibitor, FBI-1 can inhibit the activity of transcription factors, such as P53 and SP1, through direct interactions; it can ultimately suppress the expression of related tumor suppressor genes to promote tumor cell proliferation^51,52^. Previous work has indicated that FBI-1 may induce the resistance of HCC cells to antitumor agents via the P53 pathway^51,52^. However, multiple studies have indicated that P53 is often deleted and inactivated (mutated) in TNBC^53–57^, so SP1 may be the main mechanism by which FBI-1 induces TNBC cells to be resistant to olaparib.

## Supplementary information

Figure Legends of the supplemental Figures

Supplemental Table 1

Supplemental Table 2

Supplemental Table 3

Supplemental Table 4

Supplemental Figure 1

Supplemental Figure 2

Supplemental Figure 3

Supplemental Figure 4

Supplemental Figure 5

Supplemental Figure 6

Supplemental Figure 7

Supplemental Figure 8
