# Supplementary material for: FBI-1 enhanced the resistance of triple-negative breast cancer cells to chemotherapeutic agents via the miR-30c/PXR axis
Source: Cell Death Dis. 2020 Oct 13;11(10):851. doi: 10.1038/s41419-020-03053-0 (PMC7554048; doi:10.1038/s41419-020-03053-0)
Supplement: Supplementary file 1 — Figure Legends of the supplemental Figures [file 41419_2020_3053_MOESM1_ESM.doc]

Figure Legends of the supplemental Figures:

Supplemental Figure 1. The association between FBI-1 and factors related to the PXR pathway. The RNA level of PXR, BCRP, MDR-1, miR-30c, or FBI-1 in paired non-tumor specimens or TNBC (the paired specimens of TNBC and para-tumor) was examined by using the qPCR experiments. (A) the expression level of PXR, BCRP, MDR-1, miR-30c, or FBI-1 was shown as Scatter-plot images. (B) the results were shown as scatter-plot images. The abscissa is the expression level of FBI-1; the ordinate is the corresponding expression level of PXR, BCRP, MDR-1, or miR-30c. *P<0.05

Supplemental Figure 2. FBI-1 enhanced the expression of protein levels in PXR’s downstream multi-drug resistance-related genes, BCRP and MDR-1, in patient-derived TNBC cells. TNBC PDC No. 3 was transfected with FBI-1 vector or the siRNA of FBI-1. The protein samples were extracted and analyzed by western blot tests. The protein levels of FBI-1, BCRP, or P-GP were identified by their antibodies. The GAPDH was chosen as the loading control. The results were shown as the images of western blot or the quantitative results of the images. *P<0.05 versus control group with FBI-1 group; ^#^P<0.05 versus control group with siFBI-1 group;

Supplemental Figure 3. FBI-1 could not interact with PXR in TNBC cells. TNBC PDC No. 6 (A) or No. 3 (B) was transfected with a FLAG vector or the FLAG-FBI1 vector. Then, cells were harvested for IP (co-immunoprecipitation) experiments. The protein level of FLAG-FBI1 or PXR was identified by their antibodies. The results were shown as the images of western blot or the quantitative analysis of images.

Supplemental Figure 4. The FBI-1 repressed the activation of miR-30c promoter region containing the SP1 binding site. (A) The promoter region and the six luciferase reporters contain the six fragments of mR-30c’s promoter regions. The luciferase reporter containg the full length of miR-30c’s promoter region was named as Luc-All. The luciferase reporters containing the mutated SP1 binding site was named as: Luc-All^Mut^ or Luc-5^Mut^. (B-D) The effect of FBI-1 or siFBI-1 on the activation of the reporters were examined by the luciferase-activation examination. *P<0.05.

Supplemental Figure 5. The specificity of FBI-1’s function was confirmed by the colony-formation and qPCR experiments. (A) TNBC PDCs No. 3 cells were transfected with FBI-1, FBI-1 + miR-30c, FBI-1 + miR-30c + inhibitor or FBI-1 + miR-30c + PXR^Mut^. (B) TNBC PDC No. 6 were transfected with siFBI-1, siFBI-1 + miR-30c inhibitor or siFBI-1 + PXR^Mut^. Cells were treated with control or olaparib and for colony formation. The survival of TNBC cells were shown as the images of colonies, quantitative analysis of colonies. The RNA level of FBI-1, miR-30c or PXR was examined by qPCR and shown as mean±SD. *P<0.05.

Supplemental Figure 6. The overexpression or knockdown of FBI-1 in TNBC cell lines. The TNBC cell lines, HCC-1937 (A) or MDA-MB-436 (B), which were transfected with control, FBI-1 or siFBI-1, were harvested for the western blot analysis or qPCR. The protein level of PXR or FBI-1 was examined by their antibodies and the expression level of miR-30c was examined by qPCR. The results were shown as the images of western blot or relative RNA level (mean ± SD). P<0.05

Supplemental Figure 7. The effect of FBI-1 on miR-30c/PXR axis. The TNBC cell lines, HCC-1937 (A) or MDA-MB-436 (B), which were transfected with control, miR-30c, miR-30c + FBI-1 or siFBI-1 + miR-30c inhibitor, were harvested for the western blot analysis or qPCR. The protein level of PXR or FBI-1 was examined by their antibodies and the expression of miR-30c was examined by qPCR. The results were shown as the images of western blot or relative RNA level (mean ± SD). P<0.05

Supplemental Figure 8. The sequences of miR-30c1 and miR-30c2.
