# Supplementary material for: FBI-1 enhanced the resistance of triple-negative breast cancer cells to chemotherapeutic agents via the miR-30c/PXR axis
Source: Cell Death Dis. 2020 Oct 13;11(10):851. doi: 10.1038/s41419-020-03053-0 (PMC7554048; doi:10.1038/s41419-020-03053-0)
Supplement: Supplementary file 2 — Supplemental Table 1 [file 41419_2020_3053_MOESM2_ESM.doc]

Supplemental Table 1 Overexpression of FBI-1 enhanced the resistance of TNBC cells to olparib

| Cell Lines | control | FBI-1 | ketoconazole + FBI-1 |
| --- | --- | --- | --- |
|  | *IC_50_* values of olparib (μmol/L) | | |
| PDC No. 3 | 0.94±0.10 | 8.75±1.00* | 0.63±0.11 |
| PDC No. 9 | 0.85±0.33 | 9.88±0.65* | 1.64±0.52 |

The TNBC cells (PDC No. 3 and No. 9) which were transfected with plasmids were treated indicated concentration of agents. The antitumor effect of olaparib on TNBC cells was shown as the *IC_50_* values (mean±SD). *P<0.05 versus control group with FBI-1 group; *P<0.05 versus FBI-1 group with FBI-1 + ketoconazole group. with Abbreviation: TNBC, triple negative breast cancer; PDC: patients-derived cells
