# Supplementary material for: FBI-1 enhanced the resistance of triple-negative breast cancer cells to chemotherapeutic agents via the miR-30c/PXR axis
Source: Cell Death Dis. 2020 Oct 13;11(10):851. doi: 10.1038/s41419-020-03053-0 (PMC7554048; doi:10.1038/s41419-020-03053-0)
Supplement: Supplementary file 3 — Supplemental Table 2 [file 41419_2020_3053_MOESM3_ESM.doc]

Supplemental table 2 Knockdown of FBI-1 enhanced the sensitivity of TNBC cells to olparib

| Cell Lines | control | siFBI-1 |
| --- | --- | --- |
|  | *IC_50_* values of olparib (μmol/L) | |
| PDC No. 3 | 0.94±0.10 | 0.10±0.05* |
| PDC No. 2 | 1.32±0.25 | 0.22±0.02* |
| PDC No. 6 | 1.74±0.82 | 0.25±0.04* |
| PDC No. 7 | 2.04±0.77 | 0.33±0.10* |

The TNBC cells (PDC No. 3 and No. 9) which were transfected with plasmids were treated indicated concentration of agents. The antitumor effect of olaparib on TNBC cells was shown as the *IC_50_* values (mean±SD). *P<0.05 versus control group with siFBI-1 group. Abbreviation: TNBC, triple negative breast cancer; PDC: patients-derived cells
