# Supplementary material for: FBI-1 enhanced the resistance of triple-negative breast cancer cells to chemotherapeutic agents via the miR-30c/PXR axis
Source: Cell Death Dis. 2020 Oct 13;11(10):851. doi: 10.1038/s41419-020-03053-0 (PMC7554048; doi:10.1038/s41419-020-03053-0)
Supplement: Supplementary file 4 — Supplemental Table 3 [file 41419_2020_3053_MOESM4_ESM.doc]

Supplemental Table 3 FBI-1 accelerated the metabolism or the clearance of olaparib in TNBC cell lines via modulating miR-30c/PXR axis

| Groups | HCC-1937 | MDA-MB-436 |
| --- | --- | --- |
|  | half life values of olaparib (t_1/2_, hours) | |
| control | 31.22±4.67 | 28.88±4.32 |
| FBI-1 | 9.44±0.57* | 13.36±2.75* |
| siFBI-1 | 50.21±5.62* | 47.32±7.42* |
| miR-30c | 68.31±13.44* | 51.28±7.14* |
| miR-30c + PXR^Mut^ | 8.55±0.30* | 11.15±4.33* |
| FBI-1 + miR-30c | 57.26±8.91* | 44.10±5.75* |
| siFBI-1 + miR-30c inhibitor | 18.57±4.08* | 20.33±5.48* |

Table Legend: The TNBC cells lines (HCC-1937 or MDA-MB-436) which were transfected with plasmid were treated with olaparib for 12h. Then, cells were harvested and the amount of olaparib sustaining in cells were examined by the LC-MS/MS methods. The half-life values of olaparib was shown as the mean±SD. *P<0.05 versus control group with FBI-1 group; *P<0.05 versus control group with siFBI-1 group; *P<0.05 versus control group with miR-30c group; *P<0.05 versus control group with miR-30c + PXR^Mut^ group; *P<0.05 versus control group with miR-30c + FBI-1 group; *P<0.05 versus control group with siFBI-1 + miR-30c inhibitor group; Abbreviation: TNBC, triple negative breast cancer; LC-MS/MS: liquid chromatograph mass spectrometer mass spectrometer
