# Supplementary material for: FBI-1 enhanced the resistance of triple-negative breast cancer cells to chemotherapeutic agents via the miR-30c/PXR axis
Source: Cell Death Dis. 2020 Oct 13;11(10):851. doi: 10.1038/s41419-020-03053-0 (PMC7554048; doi:10.1038/s41419-020-03053-0)
Supplement: Supplementary file 5 — Supplemental Table 4 [file 41419_2020_3053_MOESM5_ESM.doc]

Supplemental Table 4. FBI-1 resistance of TNBC cell lines, HCC-1937 or MDA-MB-436, to olaparib via modulating miR-30c/PXR axis

| Groups | HCC-1937 | MDA-MB-436 |
| --- | --- | --- |
|  | The *IC_50_* values (μmol/L) of olaparib | |
| control | 0.90±0.33 | 0.79±0.28 |
| FBI-1 | 3.70±0.38* | 2.46±0.79* |
| siFBI-1 | 0.13±0.08* | 0.14±0.01* |
| miR-30c | 0.10±0.05* | 0.10±0.03* |
| miR-30c + PXR^Mut^ | 4.90±0.82* | 2.75±0.40* |
| FBI-1 + miR-30c | 0.24±0.04* | 0.21±0.02* |
| siFBI-1 + miR-30c inhibitor | 2.21±0.51* | 1.82±0.31* |

Table Legend: The TNBC cells lines (HCC-1937 or MDA-MB-436) which were transfected with plasmid were treated with indicated concentrations of olaparib. The antitumor effect of olaparib on TNBC cells was shown as the *IC_50_* values (mean±SD). *P<0.05 versus control group with FBI-1 group; *P<0.05 versus control group with siFBI-1 group; *P<0.05 versus control group with miR-30c group; *P<0.05 versus control group with miR-30c + PXR^Mut^ group; *P<0.05 versus control group with miR-30c + FBI-1 group; *P<0.05 versus control group with siFBI-1 + miR-30c inhibitor group; Abbreviation: TNBC, triple negative breast cancer;
