# Supplementary figures and images for: FBI-1 enhanced the resistance of triple-negative breast cancer cells to chemotherapeutic agents via the miR-30c/PXR axis
Source: Cell Death Dis. 2020 Oct 13;11(10):851. doi: 10.1038/s41419-020-03053-0 (PMC7554048; doi:10.1038/s41419-020-03053-0)

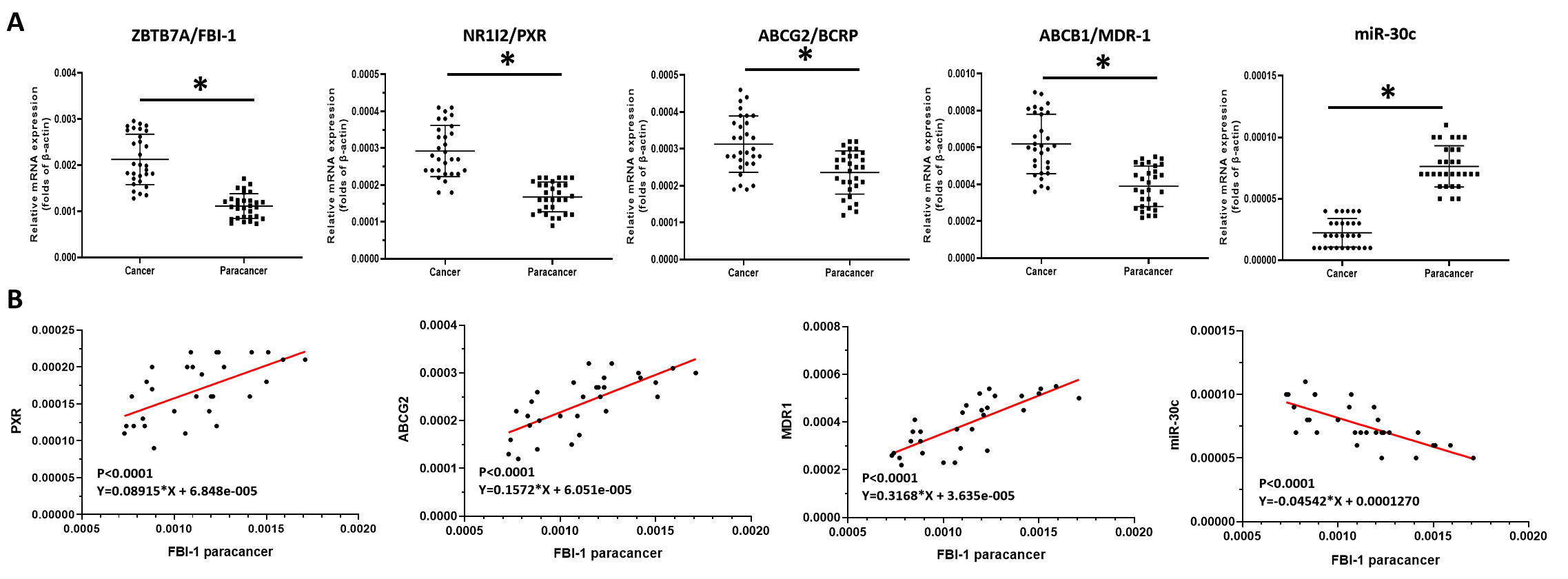

Supplement: Supplementary file 6 — Supplemental Figure 1 [file 41419_2020_3053_MOESM6_ESM.jpg]

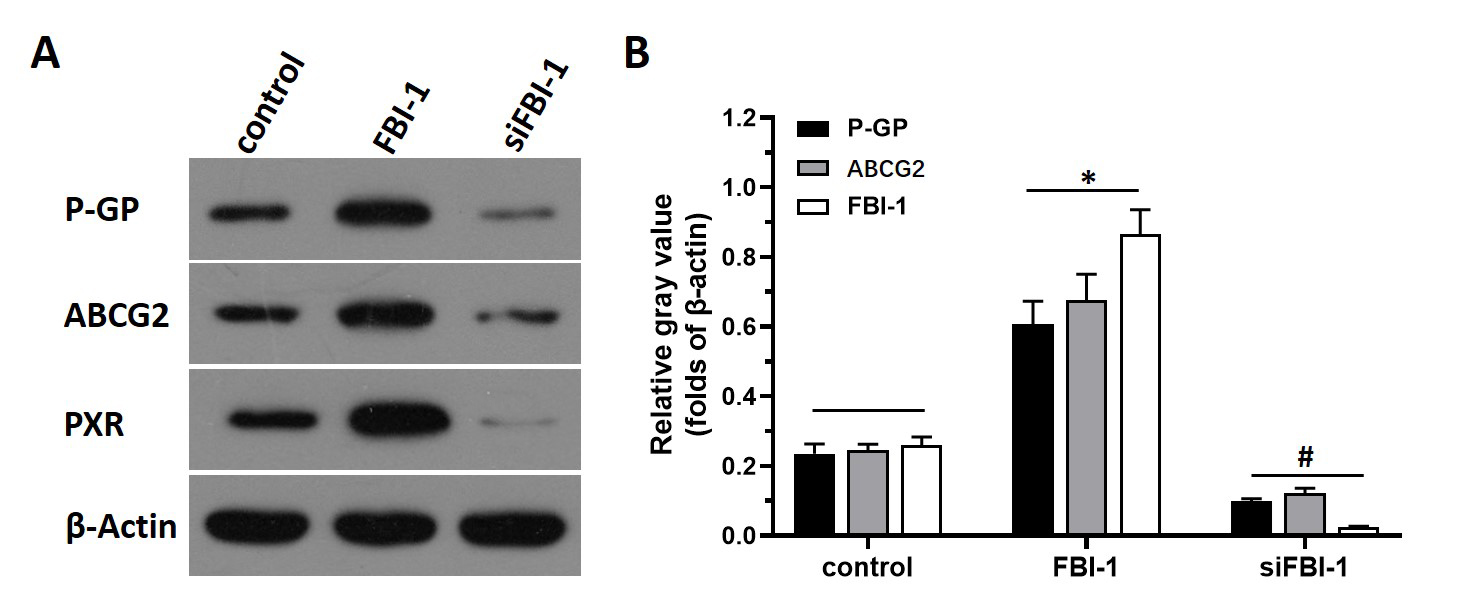

Supplement: Supplementary file 7 — Supplemental Figure 2 [file 41419_2020_3053_MOESM7_ESM.jpg]

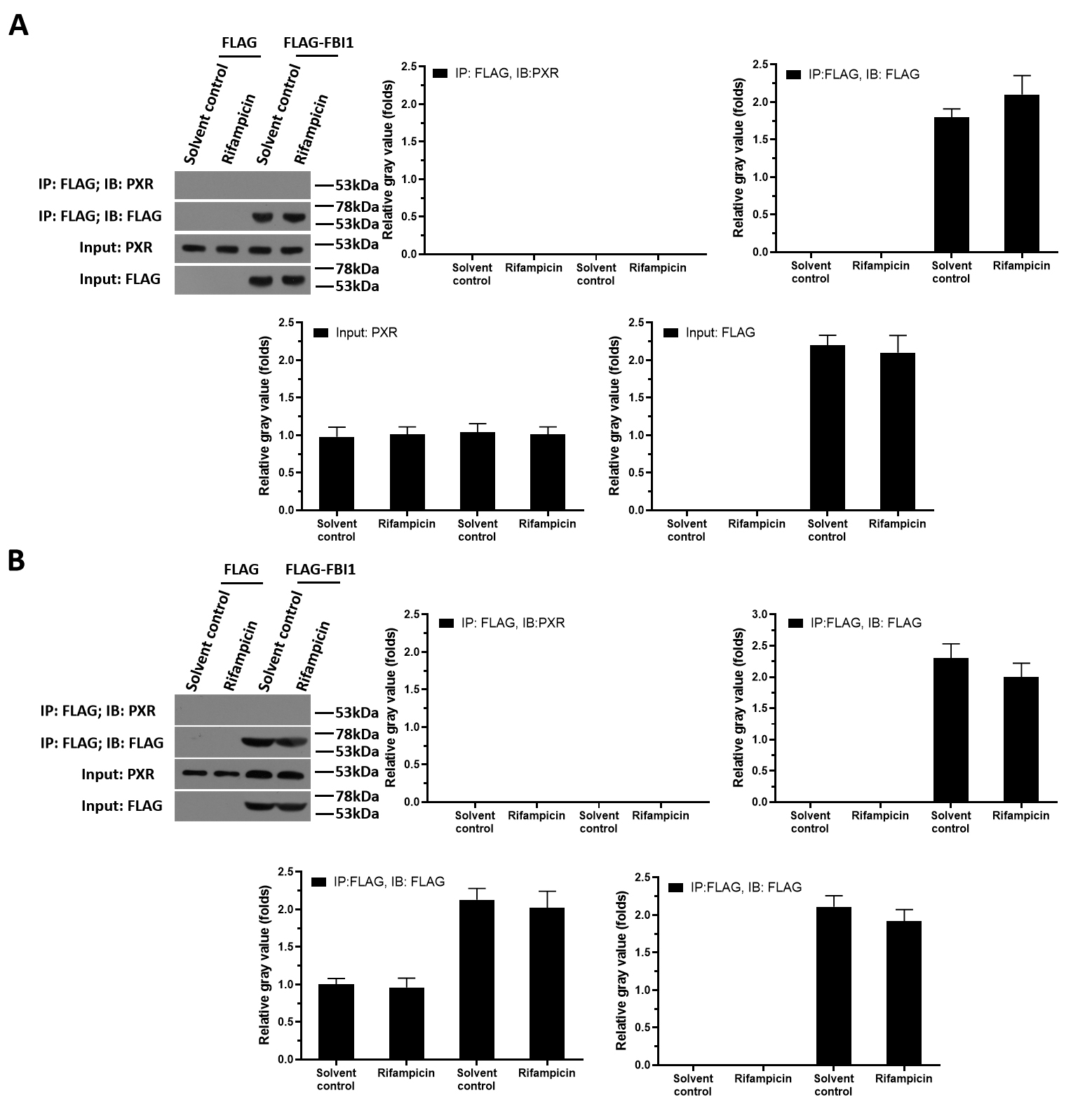

Supplement: Supplementary file 8 — Supplemental Figure 3 [file 41419_2020_3053_MOESM8_ESM.jpg]

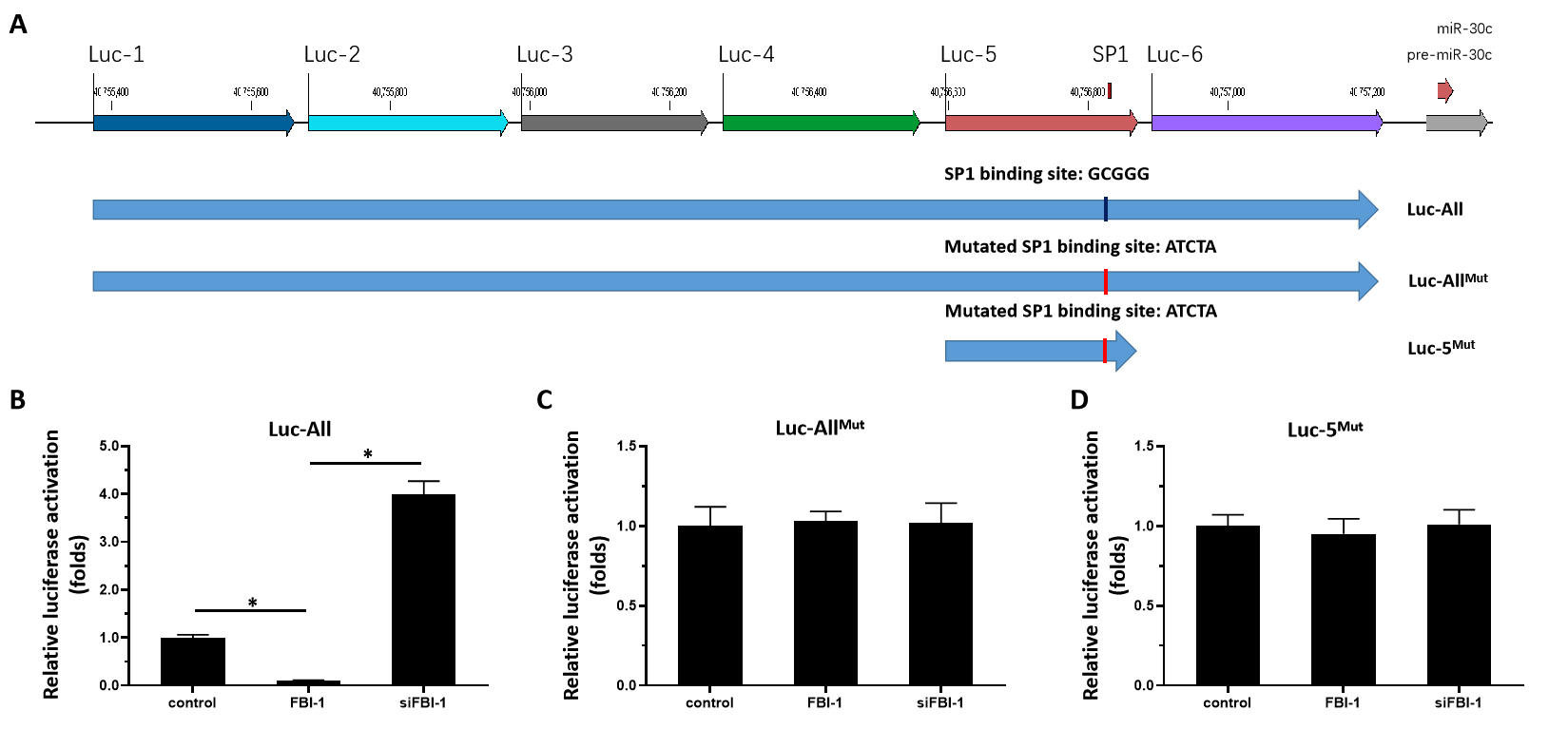

Supplement: Supplementary file 9 — Supplemental Figure 4 [file 41419_2020_3053_MOESM9_ESM.jpg]

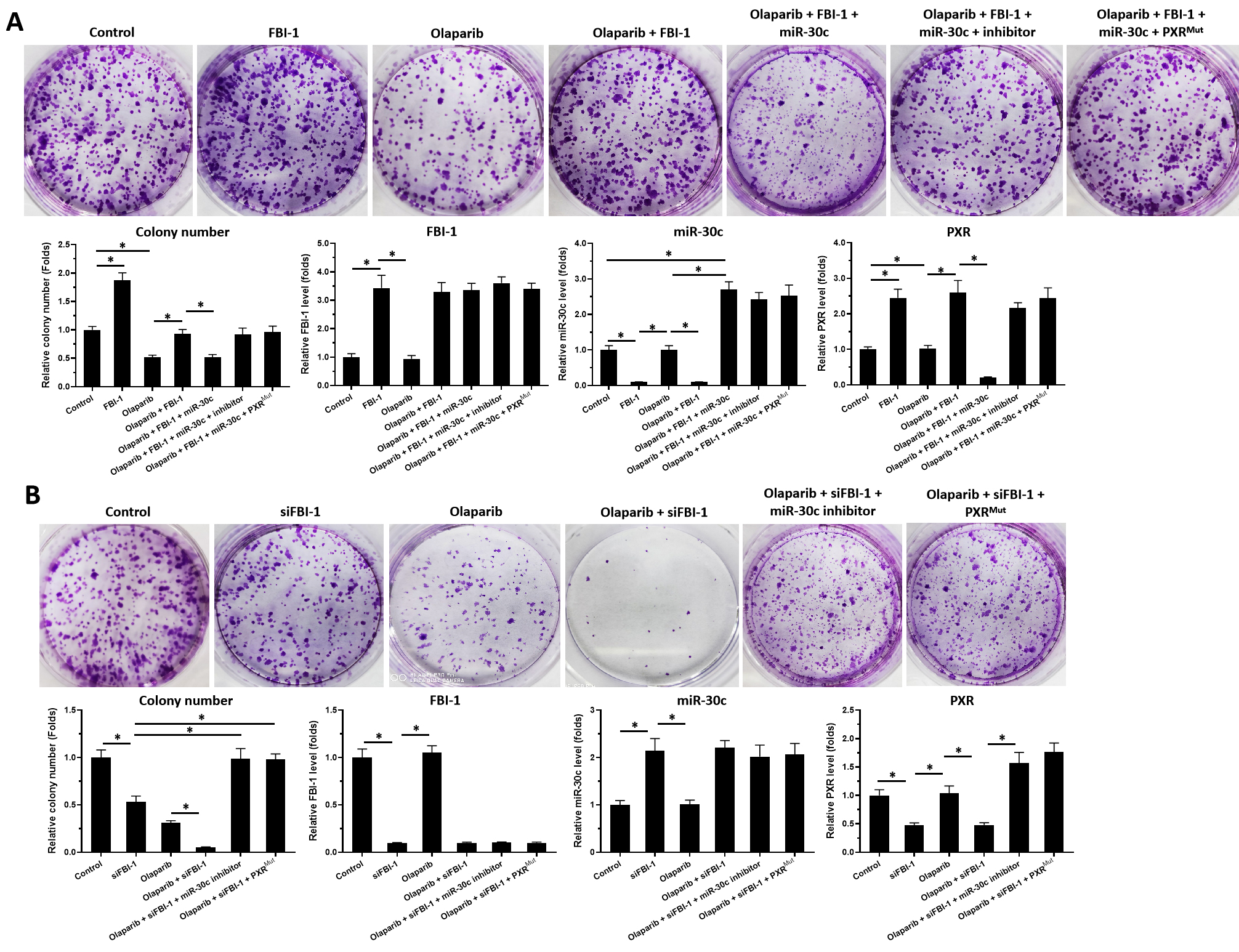

Supplement: Supplementary file 10 — Supplemental Figure 5 [file 41419_2020_3053_MOESM10_ESM.jpg]

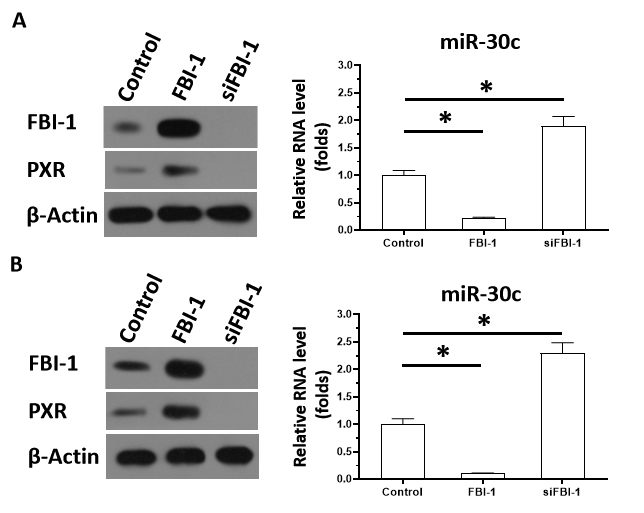

Supplement: Supplementary file 11 — Supplemental Figure 6 [file 41419_2020_3053_MOESM11_ESM.jpg]

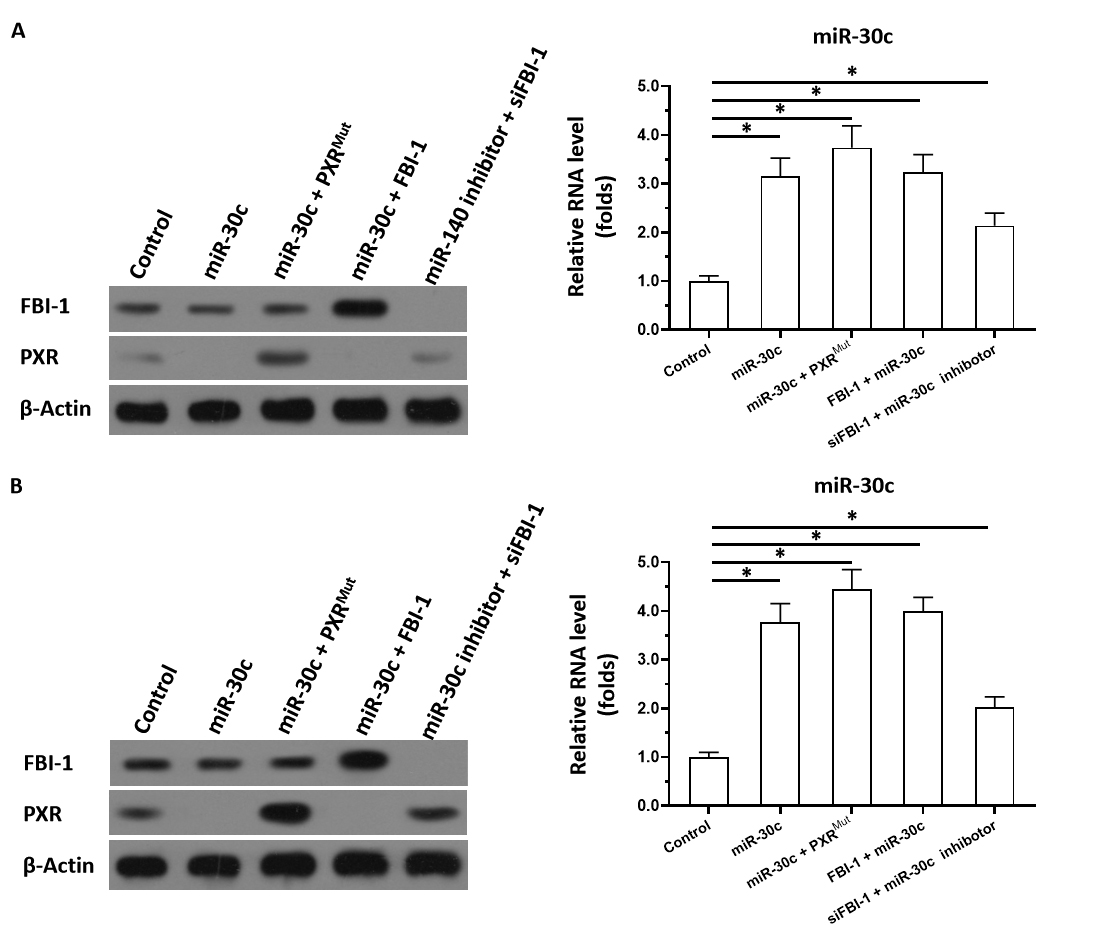

Supplement: Supplementary file 12 — Supplemental Figure 7 [file 41419_2020_3053_MOESM12_ESM.jpg]

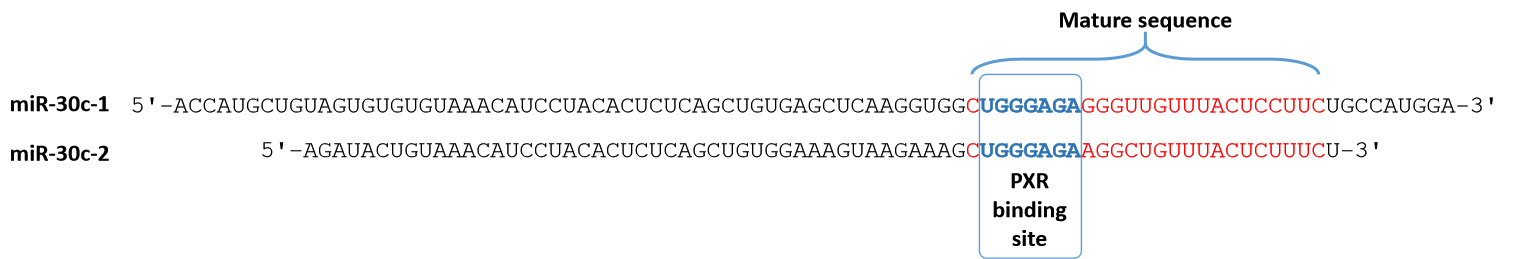

Supplement: Supplementary file 13 — Supplemental Figure 8 [file 41419_2020_3053_MOESM13_ESM.jpg]
